# Comprehensive Proteomic Profiling of *Alternaria gansuense* Provides Insights into Candidate Virulence-Associated Proteins and Core Physiological Features

**DOI:** 10.3390/cimb48080818

**Published:** 2026-08-12

**Authors:** Huaqi Liu, Lili Zhang, Tongtong Wang, Yanzhong Li

**Affiliations:** State Key Laboratory of Herbage Improvement and Grassland Agro-Ecosystems, College of Pastoral Agriculture Science and Technology, Engineering Research Center of Grassland Industry, Ministry of Education, Lanzhou University, Lanzhou 730020, China; liuhq2023@lzu.edu.cn (H.L.); zhanglili21@lzu.edu.cn (L.Z.); wangtt2023@lzu.edu.cn (T.W.)

**Keywords:** *Alternaria gansuense*, *Astragalus adsurgens*, proteomics, data-independent acquisition, pathogenicity-related proteins

## Abstract

Although *Alternaria gansuense* causes yellow stunt and root rot (YSRR)—a destructive disease of the leguminous forage *Astragalus adsurgens* in northern China—systematic investigations of this pathogen at the protein level remain scarce. In this study, we establish an optimized proteomic workflow for *A. gansuense* by comparing two protein extraction methods. Using data-independent acquisition (DIA) mass spectrometry with a DIA-NN search against the *Alternaria* protein database, we construct the first comprehensive proteome reference map of *A. gansuense*, then perform functional annotation via Gene Ontology, KOG, KEGG, InterPro domain, and subcellular localization analyses. A total of 5052 proteins were identified from vegetative mycelia, and the proteome was found to be predominantly composed of proteins involved in primary metabolism, signal transduction, secondary metabolism, and stress responses. Notably, a set of putative pathogenicity-associated proteins, including two-component regulators (SSK1p), protein kinases, cytochrome P450 enzymes, and ABC transporters, was identified. These proteins are homologs of well-characterized virulence factors in other pathogenic fungi, suggesting a potential coordinated signaling—metabolism—defense network that may contribute to fungal virulence. Subcellular localization further shows that cytoplasmic and nuclear proteins together account for over 50% of the annotated proteome. This study presents the first comprehensive proteomic reference map for *A. gansuense*, providing a valuable resource for functional genomics. Subsequent experimental validation of the bioinformatically predicted candidate molecular targets presented here may help to dissect the pathogenic mechanisms of YSRR and enable the development of novel disease management strategies.

## 1. Introduction

Standing Milkvetch (*Astragalus adsurgens* Pall.) is a perennial legume distributed throughout North America and Eurasia [1,2], which is widely cultivated in China as a high-yield forage with high protein content and good nutritional value [3]. It is also drought- and cold-tolerant, and so is often used for windbreaks and sand fixation in ecological restoration efforts. In some countries, including the United States, it is considered a toxic herb (locoweed) and a potential hazard to livestock. However, in China, many studies have reported its low toxicity, and it is generally considered safe for ruminants [4].

Large areas of artificial grassland in northern China once suffered severe degradation, the cause of which remained unclear for a long time [3,5]. In 2007, a new disease of *A. adsurgens* was reported [6]. Later work confirmed that its main cause was yellow stunt and root rot (YSRR), a systemic disease induced by the fungus *Embellisia astragali* [2], which was subsequently reclassified as *Alternaria gansuense* due to taxonomic revisions based on Woudenberg’s classification [7,8,9].

This fungus invades both the aboveground and underground tissues, causing leaf chlorosis, stem necrosis, stunting, root rot, and often whole-plant death [2]. Field surveys have reported disease incidence ranging from 5.7% to 92.52% and mortality from 1.5% to 71.24% in major cultivation areas, resulting in major yield losses [2,10]. *A. gansuense* grows slowly in vitro, and phylogenetic analyses place it close to legume-associated endophytes of *Undifilum* [11,12,13]. Infected host tissues have also been shown to contain hepatotoxic metabolites and bioactive flavonoid derivatives [6,14,15].

Proteomic approaches are essential for understanding pathogenic regulatory networks at the protein level [16]. In other *Alternaria* species, proteomic analyses have already provided important insights; for example, DIA-MS profiling of *A. alternata* revealed different proteomes between spores and hyphae and identified germination-related proteases as potential virulence factors [17]. Comparative proteomic analysis of infected tissues has revealed the activation of MAPK signaling and defense-related proteins during infection [18]. Studies combining genetic analyses have validated core virulence components such as cell wall-degrading enzymes, melanin biosynthetic proteins, and signaling factors [19,20,21,22]. A large-scale secretome analysis across 150 phytopathogenic fungi also showed that secreted protein repertoires are strongly linked to the fungal lifestyle [23].

Although proteomic data are abundant for fast-growing necrotrophic *Alternaria* pathogens, these models cannot be directly transferred to *A. gansuense*. This pathogen has distinct traits, including slow growth, persistent colonization, and the production of metabolites that are toxic to livestock. Therefore, its complete proteome and pathogenicity-related system remain poorly understood. DIA-based quantitative proteomics is well suited to address this gap, as it enables untargeted, large-scale identification and quantification of mycelial proteins, allowing for a better understanding of the coordinated signaling, metabolic, and detoxification networks that may underlie this pathogen’s particular infection strategy.

Therefore, this study aimed to construct the first comprehensive proteome reference map for *A. gansuense* using DIA-MS, enabling the identification of candidate pathogenicity-related proteins through broad functional annotation (biological process, molecular function, cellular component). These results facilitate in-depth profiling of the pathogen’s proteome, lay a protein-level foundation for understanding the pathogenesis of YSRR and developing control strategies for *A. adsurgens*, and supply candidate targets for antifungal drug development and the screening of novel bioactive compounds.

## 2. Materials and Methods

### 2.1. Fungal Strains and Growth Conditions

The *A. gansuense* strain used in this study was isolated from symptomatic *A. adsurgens* with obvious disease lesions. Rhizome segments (4–5 cm) were cut and rinsed with running tap water, then transferred to Petri dishes in a laminar flow hood (Suzhou Jinhua SJ–CJ–Bµ, Suzhou, China). The excised rhizome segments were first immersed in 70% ethanol for 30 s, followed by treatment in 1% sodium hypochlorite solution for 1 min, and finally rinsed three times with sterile deionized water [2]. The medullary tissue was cut longitudinally and aseptically inoculated onto PDA plates, which were then incubated in the dark at 22 °C for 7 days [24]. Pure cultures (strain LYZ0006) were obtained by transferring hyphal tips from colony margins to fresh PDA plates, with 10 replicates prepared [6]. After 14 days at 22 °C in the dark, active peripheral hyphae were selected, transferred to 2 mL sterile water, and homogenized. The homogenate was used to inoculate 250 mL Erlenmeyer flasks containing 150 mL PDB. Cultures were then incubated on an orbital shaker (Shanghai Sukun NSKY-2102C, Shanghai, China) at 150 rpm in the dark at 22 °C for 20 days [9]. Mycelia were collected via filtration, blotted dry on sterile filter paper, snap-frozen in liquid nitrogen, freeze-dried, and stored at −80 °C until further use.

### 2.2. Protein Extraction

Two precipitation methods were compared via 2-DE to optimize gel separation. The SDT lysis method was used for DIA mass spectrometry because it is more compatible with enzymatic digestion and LC-MS quantification (Supplementary Figure S1). *A. gansuense* has a dense, tough cell wall, so mycelia were first disrupted by grinding in liquid nitrogen [25]. SDT-extracted proteins were used for quality control and DIA untargeted proteomics. For 2-DE, two additional protocols—the phenol–methanol–ammonium acetate precipitation method and the TCA–acetone precipitation method—were compared in terms of protein purity and solubility.

#### 2.2.1. SDT Lysis Method

Frozen mycelia were ground in liquid nitrogen and transferred into pre-cooled tubes. SDT lysis buffer (containing 100 mM NaCl) and DTT (1/100 of buffer volume) were added, and the mixture was vortexed and sonicated on ice for 5 min. After centrifugation at 12,000× *g* for 15 min at 4 °C, the supernatant was collected and heated at 95 °C for 8–15 min, then cooled on ice for 2 min. IAM solution was then added and incubated in the dark for 1 h. Four volumes of pre-cooled acetone (−20 °C) were added to precipitate proteins, and samples were kept at −20 °C for at least 30 min. After centrifugation at 12,000× *g* for 15 min at 4 °C, the pellet was collected, washed with 1 mL pre-cooled acetone (−20 °C), centrifuged again, air-dried, and dissolved in DB buffer [26,27].

#### 2.2.2. Precipitation with Ammonium Acetate in Methanol Following Phenol Extraction

Mycelial powder (100 mg) was mixed with an equal volume of extraction buffer (0.1 M KCl, 0.5 M Tris base, 0.5 M sucrose, 50 mM EDTA, 30 mM HCl, 50 mM DTT). The mixture was then vortexed, homogenized on ice for 10 min, and ultrasonically disrupted at 4 °C for 30 min. After centrifugation at 13,000× *g* and 4 °C for 30 min, the supernatant was transferred and mixed with an equal volume of pH 8.0 phenol for 15 min at 4 °C. The upper phenolic phase was collected and re-extracted three times with the extraction buffer. Proteins were precipitated by adding five volumes of 0.1 M ammonium acetate in methanol (−20 °C) and incubating overnight at −20 °C. The precipitate was collected via centrifugation, washed several times with 0.1 M ammonium acetate in methanol, then washed twice with precooled acetone. After vacuum-drying, the pellet was dissolved in lysis buffer (9.8 M urea, 2% CHAPS, 0.5% ampholyte, 0.002% bromophenol blue) through repeated pipetting and vortexing at 28 °C. After 1 h incubation with shaking at 4 °C, insoluble material was removed via centrifugation. The supernatant was stored at −80 °C until IEF.

#### 2.2.3. Precipitation with TCA in Acetone

A total of 100 mg of freeze-dried mycelia was weighed and suspended in 10% TCA–acetone solution containing 0.07% β-mercaptoethanol, vortexed, and incubated overnight at −20 °C with intermittent mixing. The protein precipitate was collected via centrifugation and washed three times with precooled acetone containing 0.07% β-mercaptoethanol. Residual acetone was removed via freeze-drying. The pellet was dissolved in lysis buffer (9.8 M urea, 2% CHAPS, 0.5% ampholyte, 0.002% bromophenol blue) and, after removing debris by centrifugation, the protein concentration in the supernatant was measured via Bradford assay with ovalbumin as a standard [28]. Samples were stored at −80 °C until later analysis.

### 2.3. Protein Quality Test

BSA standard protein solution was prepared according to the instructions of the Bradford protein quantitative kit, with gradient concentrations ranging from 0 to 0.5 μg/μL. BSA standard protein solutions and sample solutions with different dilution multiples were added into a 96-well plate, filling each well up to a volume of 20 µL. Each gradient was repeated three times. Next, 180 µL of G250 dye solution was quickly added to each well of the plate, followed by incubation at room temperature for 5 min before performing absorbance measurement at 595 nm. The standard curve was drawn according to the absorbance of the standard protein solution, and the protein concentration of the sample was calculated. A total of 20 μg of the protein sample was loaded onto 12% SDS-PAGE gels for electrophoresis, with concentrated gel electrophoresis performed at 120 V for 20 min and separation gel electrophoresis performed at 150 V for 50 min. The gel was stained with Coomassie brilliant blue R-250 and destained until the bands were visualized clearly.

### 2.4. Two-Dimensional Electrophoresis

To evaluate and optimize protein extraction from *A. gansuense* mycelia for later mass spectrometry, two-dimensional electrophoresis (2-DE) was performed. For the first dimension, isoelectric focusing (IEF), 18 cm IPG strips (pH 3–11; GE Healthcare, Uppsala, Sweden) were used. A total of 100 µg protein was dissolved in 340 µL rehydration buffer (9.8 M urea, 2% CHAPS, 0.5% ampholyte, 0.002% bromophenol blue) and used for passive strip rehydration. The strips were focused in an Ettan IPGphor system (Amersham Biosciences, Little Chalfont, UK) at 20 °C with a constant current of 50 µA per strip. The voltage was increased from 500 to 8000 V, with a total of 15,360 V h. The program was as follows: 500 V for 3 h, 1000 V for 3 h, 8000 V for 4 h, and 8000 V for 2 h.

For the second dimension, focused strips were first equilibrated for 15 min in 10 mL equilibration buffer (50 mM Tris-HCl, pH 8.8; 6 M urea; 30% glycerol; 2% SDS; 0.002% bromophenol blue; 1% DTT). They were then equilibrated for another 15 min in the same buffer with 2.5% iodoacetamide replacing DTT. After two water rinses, the strips were placed on top of 20 cm × 24 cm vertical 12% SDS-PAGE gels. Following Görg’s method, the strips were sealed with low-melting-point agarose (25 mM Tris base, 192 mM glycine, 0.1% SDS, 0.5% agarose, 0.001% bromophenol blue) in a Tris–glycine buffer system [29]. Electrophoresis was run at 200 V until the bromophenol blue front reached the bottom. Molecular weight markers (Bio-Rad broad-range standard) were used for calibration.

**Figure 1:**
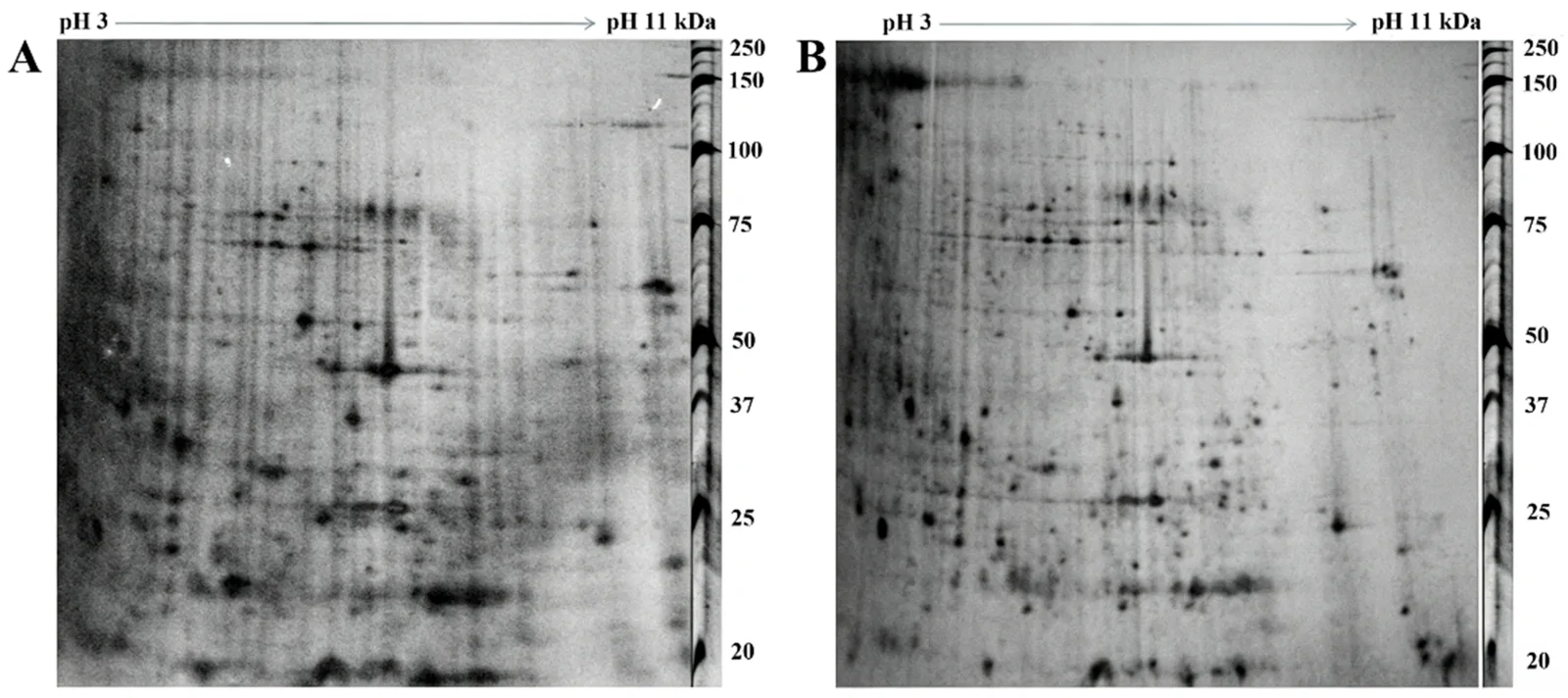
Optimization of total protein extraction protocols for *A. gansuense* via two-dimensional gel electrophoresis (2-DE). Mycelial samples were collected from three independent biological replicate cultures, processed separately with two protein extraction methods: (**A**) phenol–methanol–ammonium acetate precipitation; and (**B**) TCA–acetone precipitation. Proteins were separated with 18 cm linear pH 3–11 IPG strips in the first dimension, followed by 12% SDS-PAGE and modified silver staining. Gel images were captured with a gel imaging system. All 2-DE experiments were performed in three biological replicates; representative gel profiles are displayed. Plotting and image adjustment were completed using the ImageJ software (version 1.53t).

After fixation, protein spots were visualized using a modified silver staining method (Damerval and Shevchenko protocols) [30,31]. The gels were fixed overnight in 50% methanol and 5% acetic acid, then washed for 10 min with 50% methanol and 10 min with distilled water. They were then incubated for 1 min in 0.02% sodium thiosulfate, rinsed in water for 10 min, then immersed in 0.1% silver nitrate for 1 h at 4 °C. After another 10 min water rinse, the gels were developed in 0.04% formaldehyde in 2% sodium carbonate with vigorous shaking. Staining was stopped with 5% acetic acid, and the gels were stored in 1% acetic acid at 4 °C until analysis. All 2-DE experiments were performed in three independent biological replicates. Representative gel images are shown in Figure 1.

### 2.5. Data-Independent Acquisition Proteomic Analysis

#### 2.5.1. Trypsin Digestion and Desalting

For each protein sample, the volume was made up to 100 μL with DB lysis buffer (6 M Urea, 100 mM TEAB, pH 8.5). Then, trypsin and 100 mM TEAB buffer were added, and the sample was mixed and digested at 37 °C for 4 h. Formic acid was mixed with the digested sample, the pH was adjusted to under 3, and the solution was centrifuged at 12,000× *g* for 5 min at room temperature. The supernatant was slowly loaded into a C18 desalting column and washed with washing buffer (0.1% formic acid, 3% acetonitrile) 3 times, following which elution buffer (0.1% formic acid, 70% acetonitrile) was added. The eluents of each sample were collected and lyophilized [32].

#### 2.5.2. Vanquish™ Neo UHPLC–Astral LC/MS DIA Method

Three independent biological replicates were subjected to DIA-MS analysis, injected in the order LYZ0006_1, LYZ0006_2, LYZ0006_3. No pooled quality control (QC) samples were injected in this experiment. Each biological replicate was derived from a separate liquid culture of *A. gansuense*, inoculated and grown in parallel under identical conditions. Mycelia were harvested independently from each culture and processed separately for protein extraction, digestion, and LC-MS/MS detection. Mobile phase A (0.1% formic acid in water) and mobile phase B (0.1% formic acid in 80% acetonitrile) were prepared for separation. The lyophilized peptide powder was dissolved in mobile phase A, then centrifuged at 14,000× *g* for 20 min at 4 °C. A total of 200 ng peptide powder from each sample was loaded for analysis. A Vanquish Neo UHPLC system coupled to an Orbitrap Astral mass spectrometer was adopted, equipped with a C18 trap column (5 mm × 300 μm, 5 μm) and a PepMap TM Neo analytical column (150 µm × 15 cm, 2 μm). The whole elution gradient lasted 120 min with a constant flow rate of 300 nL/min (detailed elution gradients are listed in Table S1). Prior to batch sample injection, standard ESI tuning and mass calibration were performed on the Orbitrap Astral instrument using Thermo’s calibration mix solution, in order to guarantee stable mass accuracy. An Easy-spray ESI ion source was used, with spray voltage set to 2.0 kV and ion transfer tube temperature at 290 °C. Data-independent acquisition mode was applied for MS detection: full MS scan range *m*/*z* 380–980, primary resolution 240,000 (at *m*/*z* 200), AGC target 500%. The precursor isolation window was 2 Th, with 300 DIA windows in total, and normalized collision energy (NCE) of 25%. The secondary MS resolution was set to 80,000, and the maximum injection time was 3 ms. All raw MS data were automatically stored after acquisition.

#### 2.5.3. Protein Identification and Functional Analysis

Protein identification and quantification of the raw mass spectrometry data (.raw files) were performed using the DIA-NN software (version 1.8.1). The reference protein database used was uniprotkb_Alternaria_2026_04_13.fasta, which contains 118,102 protein sequences downloaded from UniProt for spectral matching [33]. The mass deviations of precursor and fragment ions were automatically detected and corrected by the built-in algorithm of DIA-NN. Carbamidomethylation of cysteine was set as a fixed modification, and N-terminal methionine excision was set as a variable modification. A maximum of two missed cleavage sites was allowed for tryptic digestion. For strict quality control of the identification results, a 1% false discovery rate (FDR) threshold was applied at both the peptide level (Lib.Q.Value ≤ 0.01) and protein group level (Lib.PG.Q.Value ≤ 0.01) for result filtering. Quality control analyses including peptide length distribution, precursor mass error distribution, missed cleavage rate statistics, and protein sequence coverage evaluation were performed based on the identification results. For quantitative reproducibility assessment, pairwise Pearson correlation coefficients and the coefficients of variation (CV) of protein abundance were calculated across the three biological replicates, as well as a principal component analysis (PCA). Protein quantification was performed via DIA mass spectrometry. Raw MS signal intensities were processed using the LFQ algorithm built into DIA-NN, followed by cross-sample median normalization. All protein abundance values reported throughout the manuscript refer to these normalized DIA-LFQ intensities. Gene Ontology and protein domain functional analysis were conducted using the Interproscan program against non-redundant protein databases (including Pfam, PRINTS, ProDom, SMART, ProSite, and PANTHER), while protein families and metabolic pathways were analyzed using the Diamond program against the KOG (Eukaryotic Orthologous Groups) and KEGG (Kyoto Encyclopedia of Genes and Genomes) databases [34,35].

## 3. Results

### 3.1. Construction of the A. gansuense Proteomic Reference Map

This study compared the total protein extraction efficiencies of the phenol extraction–methanol–ammonium acetate precipitation and TCA–acetone precipitation methods for *A*. *gansuense*. Proteins were separated via 2-DE and visualized via silver staining, establishing a reference 2-DE map of the proteome for this strain. Although both methods produced clear electrophoretic profiles, TCA–acetone precipitation was found to achieve superior protein separation, characterized by higher resolution of protein spots, less horizontal streaking, and a cleaner background. Therefore, this method was considered more suitable for protein sample preparation of this filamentous fungus (Figure 1).

### 3.2. Protein Quantification Results

A total of 5052 proteins were identified in this study, and all quantitative data served as the original dataset for subsequent functional annotation analyses. A comprehensive quality control assessment was performed to verify the reliability of the DIA-MS dataset.

For identification-level quality, the length distribution of identified tryptic peptides was concentrated in the range of 7–14 amino acids, consistent with the typical characteristics of tryptic digestion (Figure S2). The median precursor mass accuracy values of the three biological replicates ranged from 3.19 to 4.48 ppm, all well within the ±5 ppm tolerance window, demonstrating outstanding mass precision of the Orbitrap mass spectrometer. Statistical analysis of missed cleavage sites showed that 83.8% of peptides had no missed cleavage, indicating sufficient protein digestion. The median sequence coverage of identified proteins was 7.24%, with 21.35% of proteins showing coverage above 20%. The cumulative distribution curve of unique peptides per protein, as visualized in Figure S3, indicated robust protein identification quality.

For LC-MS/MS workflow stability, the retention time normalization profile indicated high consistency of the liquid chromatography separation system (Figure S4). To evaluate the reproducibility of the DIA-MS proteomic data, pairwise Pearson correlation coefficients and coefficients of variation (CVs) were calculated across the three independent biological replicates. The pairwise Pearson correlation coefficients all exceeded 0.94. The correlation heatmap (Figure S5) and PCA plot (Figure S6) showed that the three replicates clustered closely, indicating high consistency among independently grown samples. Statistical calculation revealed that 78% of the quantified proteins had CV values below 0.2 (Figure S7). This overall low quantitative variation profile is further visualized in the CV cumulative distribution curve and supports the reliability of the baseline proteome profile. Collectively, these QC results indicate stable and consistent performance across sample preparation, LC separation, and mass spectrometry detection. 

Detailed information on the top 20 proteins, ranked by relative abundance, is provided in Table 1. It should be noted that these quantitative data only imply basal protein abundance levels under pure culture conditions, providing a reference for subsequent studies. Functional classification revealed that highly abundant proteins were mainly associated with physiological processes including basal metabolism, redox reactions, protein folding, and translation, as well as signal transduction. 

Proteins including superoxide dismutase (A0A177DJQ0), alcohol dehydrogenase (A0A8J2N6W0), protein kinase (A0A4Q4R994), and mannitol dehydrogenase (J9Y4F0) were annotated as putative pathogenicity-associated candidates. Functional roles such as reactive oxygen species scavenging, toxin biosynthesis, signal transduction, and osmotic homeostasis have been validated for their homologous counterparts in previously published studies of other phytopathogenic fungi. While homologous functional roles have been characterized in other fungal pathogens, direct experimental evidence will be required to substantiate whether these proteins play identical infection- or stress-associated roles in *A. gansuense*.

### 3.3. GO Functional Annotation and Classification

**Figure 2:**
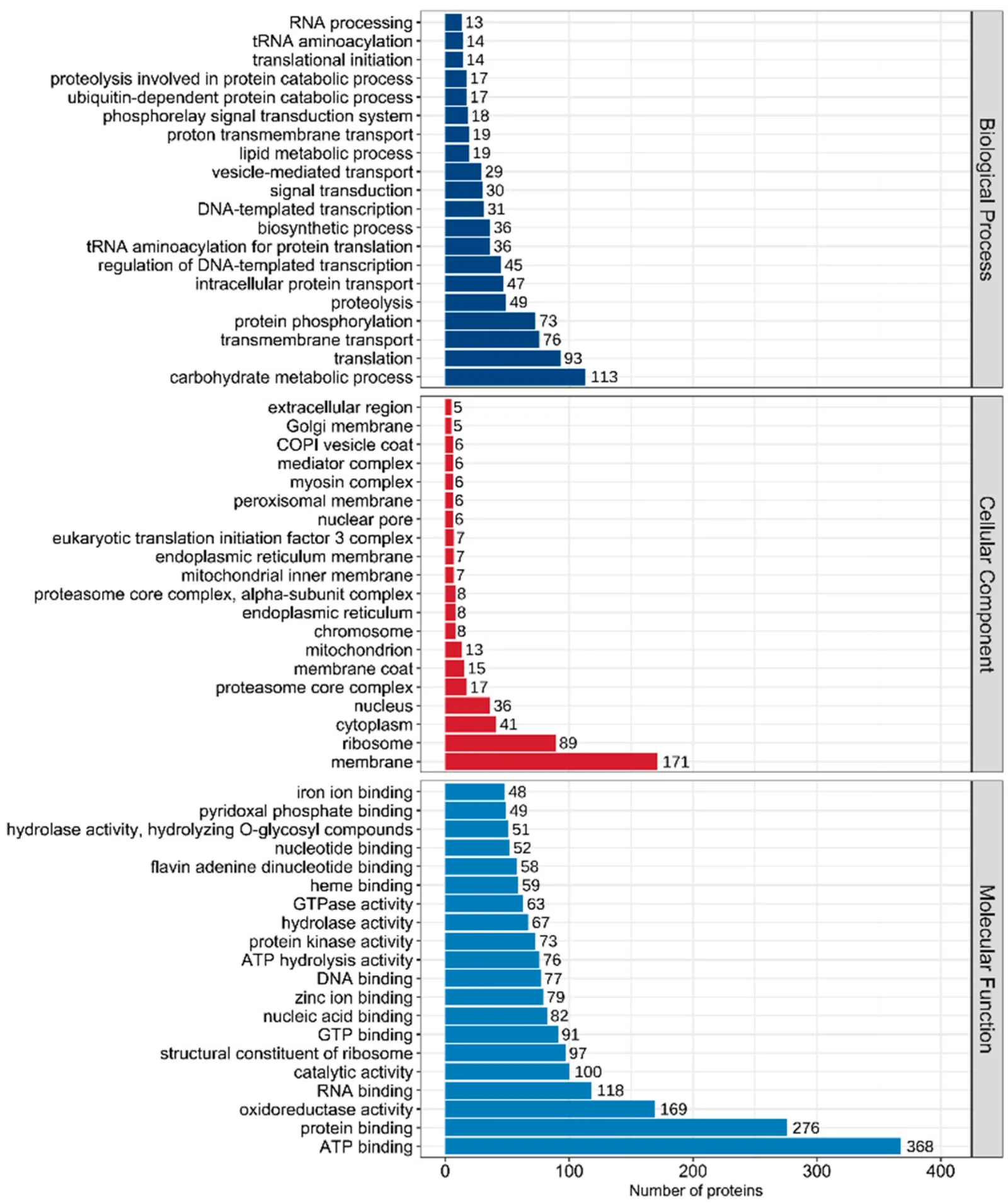
Gene Ontology (GO) functional classification of proteins identified from *A. gansuense*. Mycelia were collected from three independent biological replicates, and protein abundance values were normalized through global median normalization in DIA-NN before annotation via InterProScan. The horizontal bar plots show the top 20 most abundant secondary terms across the three GO categories: Biological Process, Cellular Component, and Molecular Function. Bar length reflects the number of matching proteins per GO entry; the x-axis indicates protein count, and the y-axis shows GO term labels. The plot was generated using R version 4.6.1 (ggplot2 package). Protein identification was filtered at FDR ≤ 0.01 at both peptide and protein levels. All data integrate results from three independent biological replicates.

Gene Ontology (GO) functional annotation was performed based on the whole-protein dataset identified via DIA mass spectrometry. Proteins were classified and statistically analyzed in the three categories of Biological Process (BP), Cellular Component (CC), and Molecular Function (MF). A total of 2758 proteins were successfully annotated with GO terms. The top 20 most annotated terms in each category are shown in Figure 2.

In the Biological Process category, the annotated proteins covered a wide range of functions. The term carbohydrate metabolic process contained the largest number of proteins (113), followed by translation (93), transmembrane transport (76), and protein phosphorylation (73). A large number of identified proteins were annotated to basic physiological and metabolic processes, including proteolysis, intracellular transport, DNA-dependent transcription, signal transduction, and vesicle-mediated transport. In contrast, relatively fewer proteins (13–19) were annotated to pathways related to nucleic acid metabolism and translational homeostasis, such as RNA processing, tRNA aminoacylation, and translation initiation. These representation patterns indicate that primary substance metabolism and genetic information translation are the core physiological activities of this strain.

According to the Cellular Component annotations, proteins were predominantly localized to membrane structures and the cytoplasm; in particular, the *membrane* category had the most annotated proteins (171), followed by ribosome (89), cytoplasm (41), and nucleus (36). Organelles and protein complexes—including endoplasmic reticulum, inner mitochondrial membrane, core proteasome complex, and nuclear pore—harbored fewer proteins (5–17). This distribution reveals that most functional proteins of the fungus reside in the cell membrane, cytoplasm, and ribosome, which is consistent with its physiological characteristics of substance transport and protein synthesis.

For the Molecular Function category, ATP binding was the most abundant term with 368 annotated proteins, followed by protein binding (276) and oxidoreductase activity (169). A large number of proteins were also annotated to the functional terms RNA binding, catalytic activity, structural constituent of ribosome, GTP binding, nucleic acid binding, and zinc ion binding (79–118). By comparison, terms associated with small molecule binding and specific hydrolase activity—including iron ion binding, pyridoxal phosphate binding, and O-glycosyl hydrolase activity—had a more moderate number of annotated proteins (48–51). Collectively, the core molecular functions of proteins in this fungus involve ATP-dependent catalysis, protein–protein interaction, and redox catalysis.

### 3.4. KOG Functional Annotation Analysis

**Figure 3:**
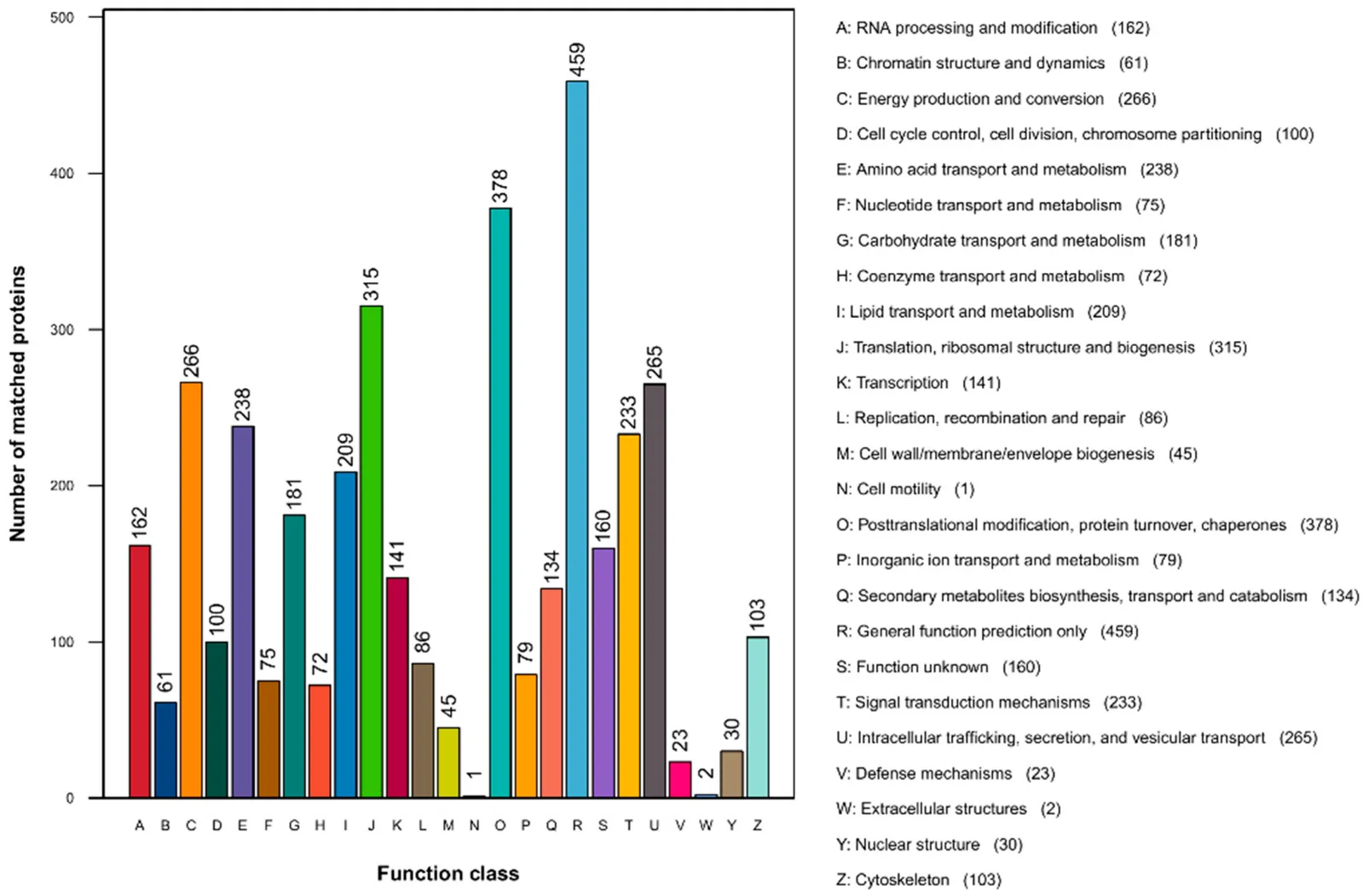
KOG functional classification of the *A. gansuense* proteome. Samples originated from three independent biological mycelial cultures; quantitative signals were normalized via global median normalization within the DIA-NN software (version 1.8.1). All proteins were annotated against the KOG database using the DIAMOND software (version 2.1.13.167). The x-axis indicates KOG functional categories, and the y-axis shows the total number of matched proteins per group. The chart was visualized with R ggplot2. The identification filtering threshold for proteins was FDR ≤ 0.01. Annotation data are aggregated from three biological replicates.

Through KOG functional classification, 3409 proteins were assigned to 26 functional subgroups (A–Z), with marked differences in protein counts across categories (Figure 3). The category of General function prediction only (R) contained the largest number of annotated proteins (459), followed by post-translational modification, protein turnover, and chaperones (O, 378) and translation, ribosomal structure, and biogenesis (J, 315). These three groups were the most highly represented functional categories in the fungal proteome. Abundant proteins were also annotated to Energy production and conversion (C, 266); Intracellular trafficking, secretion, and vesicular transport (U, 265); Amino acid transport and metabolism (E, 238); and Signal transduction mechanisms (T, 233), with protein counts ranging from 233 to 266. In addition, Lipid transport and metabolism (I, 209); Carbohydrate transport and metabolism (G, 181); RNA processing and modification (A, 162); Function unknown (S, 160); and Secondary metabolites biosynthesis, transport, and catabolism (Q, 134) accounted for relatively large proportions. In contrast, only one protein was annotated to Cell motility (N). Categories including Extracellular structures (W, 2); Defense mechanisms (V, 23); Cell wall/membrane/envelope biogenesis (M, 45); and Chromatin structure and dynamics (B, 61) harbored small numbers of annotated proteins.

### 3.5. KEGG Pathway Annotation Analysis

**Figure 4:**
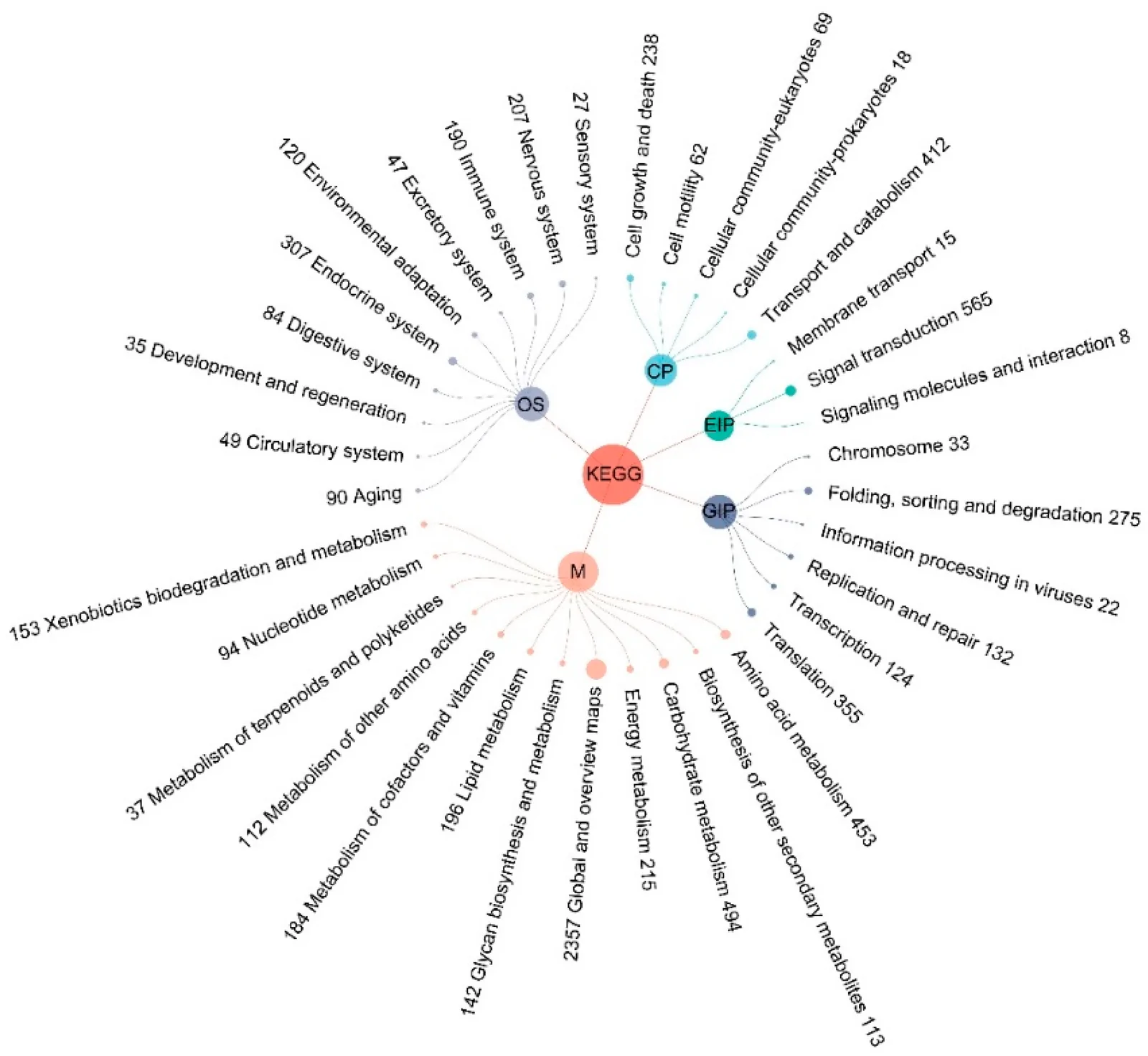
KEGG pathway hierarchical annotation of proteins detected in *A. gansuense* vegetative mycelia. Three independent biological replicate cultures were analyzed, and protein intensities were normalized via global median normalization in DIA-NN. Database alignment was performed with the DIAMOND software (version 2.1.13.167). The radial plot splits all annotated pathways into five top-level KEGG branches (M, GIP, EIP, CP, OS). The number beside each sub-pathway denotes the count of mapped proteins. Note: The Organismal Systems (OS) branch is animal-specific; the fungal proteins mapped here only perform homologous stress/growth regulatory functions without genuine animal organ systems. The figure was drawn with R ggplot2. The protein identification cutoff was FDR ≤ 0.01. Data represent combined results from three biological replicates.

Based on the results of GO functional annotation and KOG classification, we further performed pathway annotation using the Kyoto Encyclopedia of Genes and Genomes (KEGG) database to systematically dissect the biological functions of the proteins identified via DIA mass spectrometry from the perspectives of metabolic homeostasis and signal transduction. A total of 2269 proteins were assigned clear pathway information through database alignment and annotation, which fall into five primary functional categories: Metabolism, Genetic Information Processing, Environmental Information Processing, Cellular Processes, and Organismal Systems (Figure 4).

Metabolism was the primary pathway category, with the largest number of annotated proteins. Among its secondary sub-categories, global and overview maps had the highest cumulative annotation entries (2357 in total); notably, the cumulative number of entries exceeded the count of non-redundant proteins, as a single protein can be assigned to multiple KEGG pathways simultaneously. Among specific functional sub-pathways, carbohydrate metabolism (494), amino acid metabolism (453), and energy metabolism (215) ranked as the top three in terms of annotated protein numbers. Moderate numbers of proteins were also annotated to lipid metabolism, cofactor and vitamin metabolism, xenobiotics biodegradation and metabolism, and secondary metabolite biosynthesis. Collectively, these results indicate that carbon utilization, amino acid synthesis, and energy supply constitute the core metabolic activities of this fungus.

Within the Genetic Information Processing category, translation (355) and protein folding, sorting, and degradation (275) contained the most annotated proteins; transcription (124) and replication and repair (132) also presented substantial annotated protein populations, while pathways associated with chromosomal dynamics and viral genetic processing had far fewer matched proteins. This pattern demonstrates that protein synthesis, post-translational modification, and degradation are highly active in this strain.

The Environmental Information Processing category generally contained few annotated proteins, with signal transduction being the only sub-pathway with a highly represented protein population (565). By contrast, membrane transport (15) and signaling molecules and interaction (8) had extremely low protein counts, suggesting that hierarchical signal cascades serve as the core route for this fungus to sense external cues, and potentially supporting intracellular physiological responses.

In the Cellular Processes category, transport and catabolism (412) and cell growth and death (238) accounted for the majority of annotated proteins. Pathways related to eukaryotic cell communities and cell motility had relatively few annotated proteins, which is consistent with the KOG result that only one protein was annotated to cell motility.

It is critical to preface the Organismal Systems annotation results with a clear disclaimer: this KEGG top-level category was exclusively constructed for multicellular animals with differentiated organs. Fungi do not possess endocrine, nervous, immune, circulatory, or sensory organ systems, so proteins mapped to these sub-pathways do not represent true organ-specific machineries. Among the annotated entries under Organismal Systems, the sub-pathway annotated as “endocrine system” contained the largest number of proteins (307), followed by entries labeled “nervous system” (207) and “immune system” (190). These homologous fungal proteins primarily execute conserved regulatory roles in nutrient sensing, hormone-like secondary signal transduction, and reactive oxygen scavenging, rather than animal endocrine/neural immune functions. Minor sub-categories including aging, development, and regeneration had far fewer matched proteins, all of which are putatively associated with fungal vegetative growth adjustment and abiotic stress adaptation.

In summary, KEGG pathway annotation identified numerous proteins mapped to metabolism, translation, and signal transduction pathways, providing a pathway-level foundation for subsequent functional inference.

### 3.6. InterPro Conserved Domain Annotation Analysis

**Figure 5:**
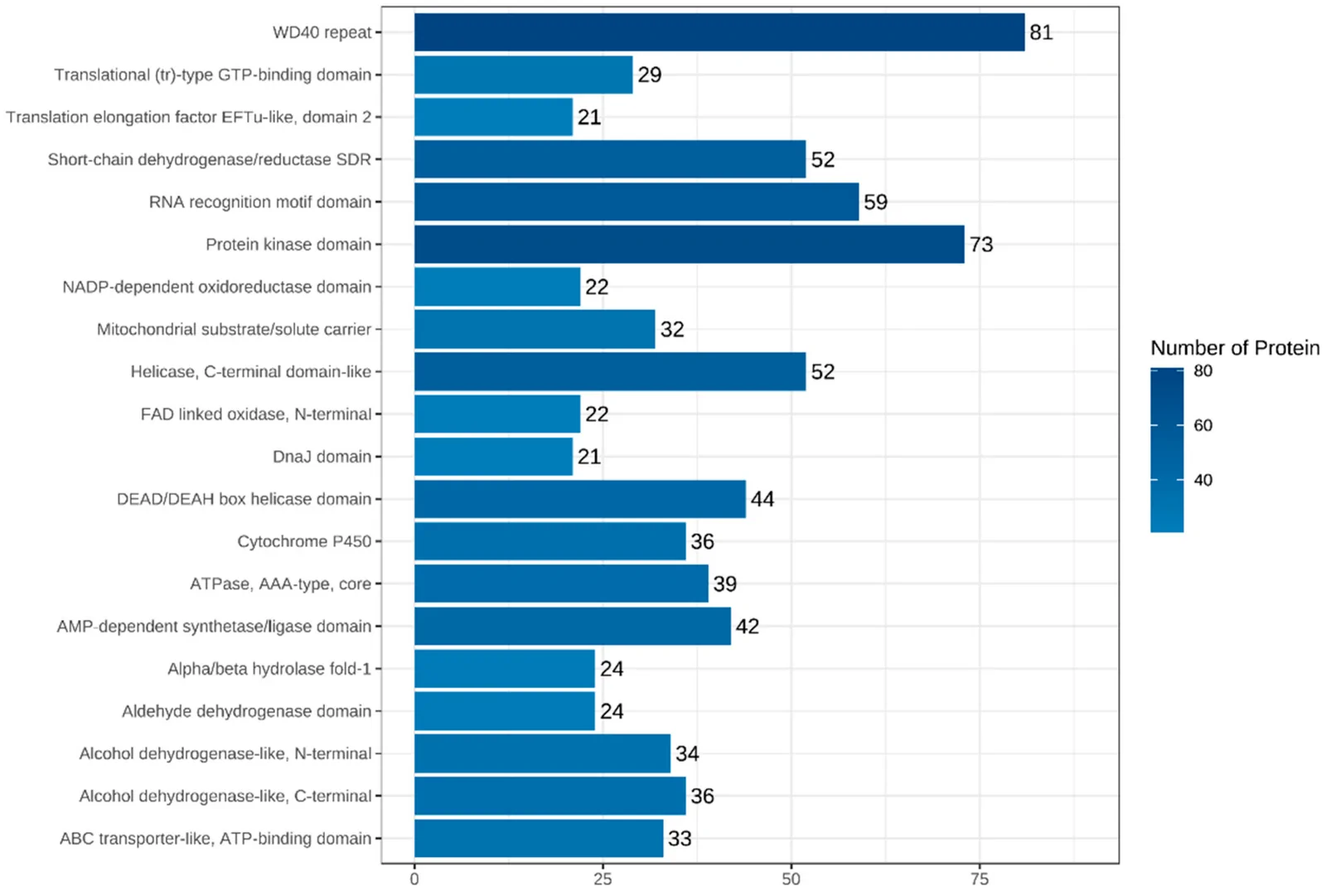
Top 20 InterPro (IPR) conserved domains in the *A. gansuense* proteome. Mycelial samples were harvested from three independent biological replicates, and protein quantification data were globally median-normalized in DIA-NN. Conserved domain annotation was conducted using the InterProScan software (version 5.72-103.0). Horizontal bars show the number of proteins containing each conserved domain (x-axis = protein count, y-axis = IPR entry name). Visualization was performed using R ggplot2. All protein identifications were filtered at FDR ≤ 0.01. Data summarize the results from three biological replicates.

To further characterize protein functions at the level of conserved domains, we performed conserved domain annotation for all proteins identified via DIA using the InterPro (IPR) database on the basis of the KEGG pathway annotations. A total of 4424 proteins were successfully annotated with at least one conserved domain, and the distribution of domains ranked by the number of annotated proteins is shown in Figure 5.

The results demonstrate that the WD40 repeat was the most abundant conserved domain, matching a total of 81 proteins, followed by the protein kinase domain (73) and RNA recognition motif domain (59). The short-chain dehydrogenase/reductase (SDR) domain and helicase-like C-terminal domain were also highly represented, each with 52 proteins annotated. These highly represented domains are involved in protein interaction and assembly, phosphorylation-mediated signal regulation, RNA transcription and translation, and redox metabolism.

In addition, a considerable number of proteins were annotated to the DEAD/DEAH box helicase domain (44), AMP-dependent synthetase/ligase domain (42), core AAA-type ATPase domain (39), cytochrome P450 domain (36), C-terminal alcohol dehydrogenase-like domain (36), N-terminal alcohol dehydrogenase-like domain (34), and ABC transporter-like, ATP-binding domain (33). In contrast, fewer proteins (21–29) were matched to the GTP-binding, translation elongation factor EF-Tu, DnaJ chaperone, and FAD-dependent oxidase domains.

The distribution and abundance profiles of diverse conserved functional domains further corroborate the core physiological functions of this strain at the molecular component level. Domains including the ATP-binding domain of ABC transporters and mitochondrial solute carrier domains exhibit prominent abundance, implying active transmembrane substance transport and mitochondrial substrate exchange in this fungus. A considerable share of identified proteins carried cytochrome P450 and dehydrogenase conserved domains. Based on the conserved domain annotations and published research on fungal homologs, these domains are associated with secondary metabolite synthesis and xenobiotic detoxification functions. It should be emphasized that the corresponding metabolic capacities of *A. gansuense* were merely inferred from sequence features and not directly measured in this study. Furthermore, the widespread occurrence of helicase, RNA-binding, and translation-associated functional domains reinforces the conclusion that processes governing genetic information replication, transcription, and translational modification are highly active within this fungus.

In summary, the InterPro conserved domain annotations provide supplementary functional information at the protein structural level. Together with the GO, KOG, and KEGG annotation results, a more comprehensive analysis of the core functional characteristics of this strain could be conducted.

### 3.7. Protein Subcellular Localization and Spatial Distribution Analysis

**Figure 6:**
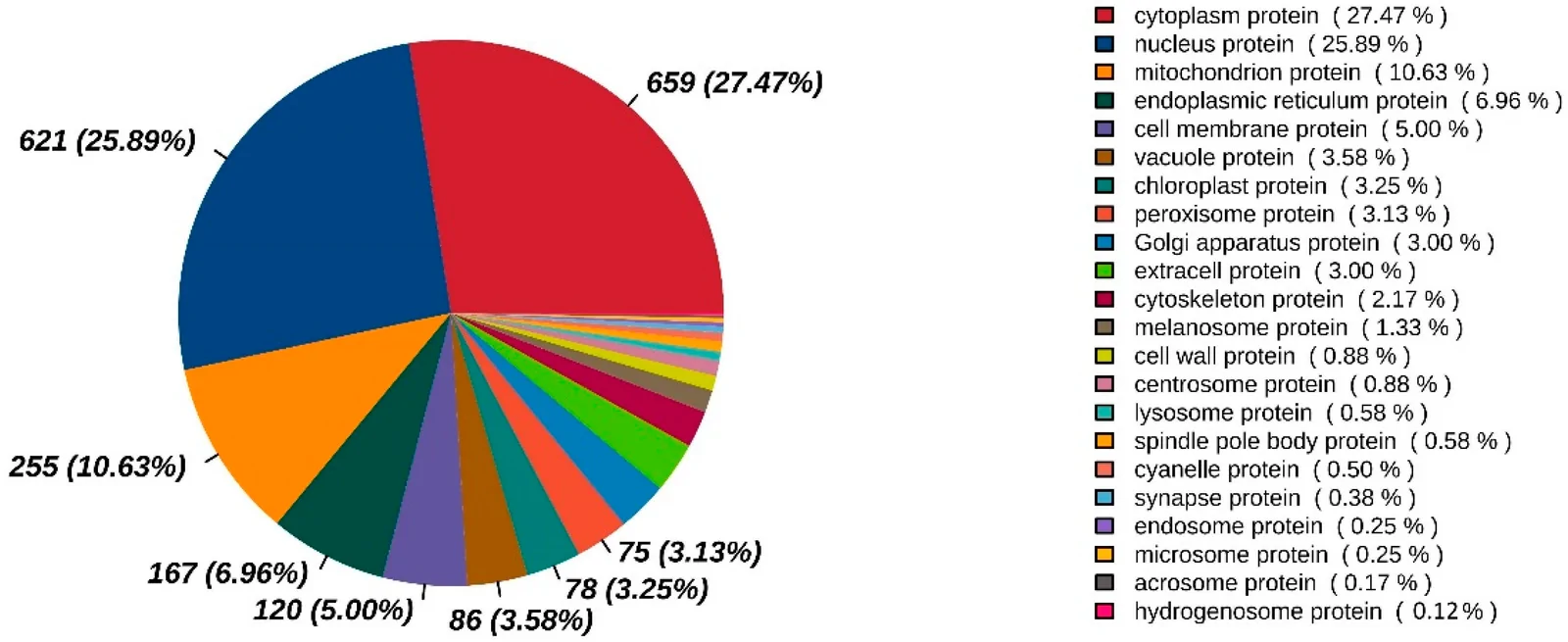
Subcellular spatial distribution of annotated proteins from *A. gansuense*. Samples were collected from three independent biological replicate cultures, with protein intensities normalized via global median normalization in DIA-NN. Subcellular localization prediction was carried out using Cell-mPLoc 2.0. Each colored sector corresponds to one cellular compartment; numbers and percentages show the proportion of annotated proteins assigned to each location. Note: Chloroplast-labeled proteins are homologous mitochondrial/cytosolic enzymes which were misassigned by the prediction software, as fungi lack chloroplasts. The pie chart was generated using R ggplot2. The protein identification threshold was FDR ≤ 0.01. All localization data integrate three biological replicates.

In this study, a subcellular localization analysis was performed to further dissect the functional characteristics of the whole proteome of the considered fungus from a spatial distribution perspective. The sequences of all identified proteins were aligned against the reference database using the Diamond software, and subcellular localization prediction was implemented via the Cell-mPLoc 2.0 database. A total of 2399 proteins were successfully annotated with localization information (Figure 6).

Statistical analysis indicated that cytoplasmic proteins were the most abundant, with 659 members accounting for 27.47% of all annotated proteins, followed by nuclear proteins (621, 25.89%); collectively, these two groups accounted for more than half of the annotated proteome. Our subcellular localization data suggest that the cytoplasm and nucleus accommodate the bulk of the fungus’s functional proteins. Predicted functions linked to these subcellular regions include central physiological events such as material metabolism, gene transcription, and translational regulation; however, it should be noted that these functional assignments rely entirely on bioinformatic annotation and require additional experimental verification. This distribution pattern is highly consistent with the previous GO Cellular Component results showing abundant proteins associated with the cytoplasm, ribosomes, and nucleus, as well as the large number of proteins annotated to Genetic Information Processing pathways in the KEGG analysis.

Mitochondrial proteins ranked third (255, 10.63%). As mitochondria serve as the primary site of cellular energy metabolism, the high proportion of detected mitochondrial proteins is consistent with the large number of proteins mapped to energy metabolism pathways in our KEGG functional analysis. Endoplasmic reticulum proteins (167, 6.96%) and cell membrane proteins (120, 5.00%) also presented relatively high proportions, suggesting active protein folding, processing, and transmembrane transport in this strain. This distribution pattern is in line with the representation of membrane proteins and transport-related metabolic pathways in the GO analysis.

Vacuole-localized proteins (86, 3.58%), peroxisome-localized proteins (75, 3.13%), chloroplast-localized proteins (78, 3.25%), Golgi apparatus-localized proteins, and extracellular secreted proteins (3.00% each) exhibited moderate proportions. Proteins targeted to other cellular structures, including the cytoskeleton, cell wall, lysosome, and centrosome, presented relatively low abundance (all accounting for less than 3%). Notably, fungi do not contain chloroplasts, and the proteins annotated as chloroplast-localized are mainly conserved energy metabolism enzymes and ribosomal proteins of endosymbiotic origin, which were misassigned by the prediction tool due to their high sequence homology with plant chloroplast proteins and are actually localized to the mitochondria or cytoplasm in fungal cells. Such discrepancy is a common bias of prediction tools, and the observed proportion is consistent with findings from published fungal proteomic studies. 

In conclusion, the subcellular localization analysis supplemented the functional information of proteins with information on their spatial distribution. Combined with the functional annotation results presented above, this helped to establish a multi-dimensional framework for comprehensive proteomic functional analysis of this strain.

## 4. Discussion

### 4.1. Methodological Considerations for A. gansuense Proteomics

The rigid chitin-rich cell wall of filamentous fungi creates major obstacles for high-quality protein extraction—a technical bottleneck which has been widely reported in proteomic studies of *Alternaria* and *Fusarium* pathogens. Consistent with previous work on other plant pathogenic fungi, our 2-DE comparison demonstrated the superior performance of TCA–acetone precipitation over phenol-based extraction, largely due to its efficient removal of polysaccharide and pigment contaminants that disrupt isoelectric focusing [36,37]. For quantitative DIA-MS, the SDT lysis protocol was selected due to its high protein solubilization efficiency and compatibility with tryptic digestion, outperforming conventional precipitation methods for bottom-up proteome quantification.

Unlike most published fungal proteomic pipelines, which typically only adopt a single extraction strategy, this study established a dual complementary workflow tailored to the unique tough mycelial characteristics of the slow-growing *A. gansuense*: TCA–acetone precipitation optimized for gel-based separation, in addition to SDT lysis for high-throughput DIA quantification. This combined protocol provides a standardized reference for proteomic sample preparation of other hard-to-lyse filamentous fungi with melanized cell walls.

Global median normalization, as implemented in DIA-NN, effectively eliminated systematic deviations from sample loading and instrument response, yielding highly reproducible quantitative datasets supported by tight replicate correlations and low CV values. The consistent annotation trends across the GO, KOG, KEGG, and InterPro databases, all converging on primary metabolism, protein translation, signal transduction, and transport/detoxification, further verify the reliability of this proteomic resource, laying a solid foundation for the prediction and subsequent functional validation of candidate pathogenicity-associated proteins in this fungus.

### 4.2. General Physiological Characteristics of the Proteome of A. gansuense

Protein quantification was performed using normalized DIA-LFQ intensities generated via DIA-NN, and the top 20 most abundant proteins from axenic mycelia are summarized in Table 1. Among these highly expressed proteins, only 1 entry (A0A4Q4NIN8) lacks clear functional annotation in the UniProt database; the remaining 19 correspond to well-characterized enzymes and stress-related factors with conserved homologs across filamentous fungi. We classified these abundant proteins into four functional clusters in order to dissect their biological relevance to the unique physiology of the slow-growing, persistently parasitic fungus *A. gansuense*.

First, antioxidant and stress-adaptive proteins occupy multiple high-abundance positions, including Cu/Zn superoxide dismutase (A0A177DJQ0), small heat-shock protein (A0A4QNHQ7), alcohol dehydrogenase (A0A8J2N6W0), and NADP-dependent mannitol dehydrogenase (J9Y4F0). SOD functions as the primary ROS scavenger to counteract plant oxidative bursts during early colonization, while mannitol dehydrogenase drives mannitol synthesis, a key compatible solute for osmotic and nutrient stress tolerance. Unlike fast necrotrophic *Alternaria* species, which only upregulate these proteins upon host exposure, their constitutive extreme accumulation in pure culture represents a core adaptive trait of *A. gansuense*. This pre-synthesized antioxidant pool allows the fungus to immediately withstand host-derived reactive oxygen stress upon initial contact with *A. adsurgens*, matching its long-term persistent colonization strategy.

Second, core carbon and energy metabolism enzymes, such as glyceraldehyde-3-phosphate dehydrogenase, enolase, and pyruvate decarboxylase, were highly enriched in the abundance list. These enzymes constitute the backbone of glycolysis and fermentative pathways, supporting continuous vegetative expansion even under low-nutrient axenic conditions. The sustained high expression of glycolytic machinery correlates with the pathogen’s slow growth and thick melanized cell wall, which imposes high energy demands for hyphal elongation and cell wall remodeling.

Third, translational regulatory proteins, including elongation factor 1α and translation initiation factor 5A, were prominent among abundant hits. Robust translation machinery guarantees rapid de novo synthesis of signaling and degrading enzymes once the fungus invades host tissues, which explains the constitutive basal expression of virulence-related kinases and CAZymes (as highlighted in Section 4.3).

Notably, the most abundant protein A0A4Q4NIN8 bears a conserved GRG1 domain, according to NCBI CDD analysis. Prior proteomic profiling of *Aspergillus fumigatus* conidia reported Grg1 (AFUA_5G14210) as a highly accumulated small protein with unconfirmed biological function [38]. The extraordinary constitutive abundance of this lineage-specific protein in our vegetative hyphae implies its potential involvement in sustained stress adaptation during prolonged saprophytic growth, which requires future genetic verification.

Similar core metabolic signatures of saprophytic proliferation have been documented in proteomic surveys of *A. brassicicola*, revealing a shared reliance on carbohydrate, amino acid, and energy metabolism to support vegetative expansion [39]. However, prominent and biologically meaningful physiological differences separate these two pathogens; for example, *A. brassicicola* grows rapidly and initiates rapid necrotic host–cell death post-infection, with stress-related proteins only significantly induced after exposure to host tissues. In contrast, the slow-growing *A. gansuense* constitutively accumulates large quantities of antioxidant and molecular chaperones under axenic culture, an adaptive trait suited to its persistent colonization lifestyle and tolerance to prolonged nutrient limitation.

Collectively, the overall abundance landscape reflects an adaptive physiological strategy specialized for the saprophytic and parasitic growth of *A. gansuense*, clearly distinguishing it from fast-acting necrotrophic congeneric pathogens. The overrepresentation of primary metabolic proteins supports basic hyphal proliferation, while the extreme enrichment of stress resistance factors forms a pre-emptive defense system against host antagonism [40,41]. Several high-abundance proteins (SOD, mannitol dehydrogenase) were also annotated as predicted virulence regulators, linking basal physiological homeostasis to the pathogen’s capacity for infection. Notably, motility-related proteins were found to be nearly absent from the full proteome, consistent with the universal trait of *Alternaria* fungi relying on mycelial extension and conidia dispersal, rather than flagella, for host colonization [42].

Further functional dissection of subcellular localization data revealed a set of predicted extracellular secreted proteins within the proteome, which act as central mediators of fungus–host crosstalk. Among these secreted products, carbohydrate-active enzymes (CAZymes) constitute the largest functional subset, including multiple annotated cellulases, pectate lyases, and hemicellulases [43]. These enzymes synergistically degrade the cellulose, pectin, and hemicellulose that form the rigid cell wall of *A. adsurgens*, breaking down structural polysaccharides into usable monosaccharides to support fungal nutrient acquisition during colonization [44]. Distinct from *A. brassicicola*—which drastically upregulates CAZyme secretion only upon exposure to host tissue—*A. gansuense* maintains constitutive moderate expression of diverse CAZy isoforms under axenic culture; a pre-emptive adaptive trait matching its slow, persistent parasitic lifestyle [45].

### 4.3. Putative Pathogenicity-Associated Proteins and Pathways Inferred from Functional Annotation

Before elaborating candidate virulence regulators, we emphasize that all functional interpretations within the current study are derived exclusively from homology-based bioinformatic prediction, without in planta or genetic validation. The coordinated combination of signaling, metabolic, and detoxification proteins detected in vegetative mycelia provides a preliminary hypothetical regulatory network that may support host colonization by *A. gansuense*, which can be further validated through genetic manipulation in subsequent experiments.

Two-component systems and MAPK cascades are core upstream modules for pathogenicity in *Alternaria* and other necrotrophic fungi [46]. Knockout studies in *A. alternata* and *A. brassicicola* showed that the two-component regulator SSK is required for osmotic stress adaptation, secondary metabolite production, and host penetration; in particular, mutants showed strongly reduced virulence [47]. In our dataset, the orthologous SSK1p protein, together with abundant serine/threonine protein kinases and MAPK family members, were constitutively detected under axenic pure culture. Unlike most comparative proteomic surveys of *Alternaria,* which only captured significant induction of signaling proteins after exposure to host tissues, our data reveal that these key signaling components are stably expressed at basal levels in vegetative mycelia. This suggests that *A. gansuense* maintains a pre-formed signaling reservoir, enabling rapid activation of downstream infection pathways after contact with *A. adsurgens*.

Secreted carbohydrate-active enzymes (CAZymes) represent core virulence effectors that are conserved across *Alternaria* pathogens. Transcriptomic and proteomic datasets obtained from *A. alternata* show that cellulases and pectinases remain weakly expressed under axenic culture and are sharply induced upon host contact [44]. In contrast, multiple CAZy isoforms are constitutively abundant in axenic *A. gansuense* mycelia, as supported by our secretory protein predictions and GO extracellular component annotations. This pre-formed enzyme pool enables immediate host tissue degradation upon colonization—a distinctive adaptive trait absent from fast necrotrophic congeneric species. Subcellular localization data further support this interspecific divergence: cytoplasmic and nuclear proteins dominate all filamentous phytopathogens, yet *A. gansuense* harbors a larger proportion of mitochondrial proteins, matching its slow growth and high energy demand to penetrate melanized cell walls [48].

Downstream of signal transduction, a large repertoire of redox enzymes—including cytochrome P450s and short-chain dehydrogenases/reductases (SDRs)—constitutes the robust secondary metabolic machinery. Cytochrome P450 monooxygenases have been repeatedly verified as essential virulence determinants in host-specific *Alternaria* pathotypes, where they catalyze phytotoxic secondary metabolites that damage host cells. Our previous toxicological research confirmed that *A. gansuense* infection produces swainsonine and causes liver injury in mice [14]. The broad P450 and SDR repertoire identified here provides strong evidence that this pathogen possesses intact toxin biosynthetic pathways, distinguishing it from non-toxigenic saprophytic *Alternaria* species with far fewer secondary metabolic oxidoreductases [49].

During host infection, plants trigger an oxidative burst to eliminate invading fungi via reactive oxygen species (ROS); as such, endogenous antioxidant machinery is critical for pathogen survival [50]. Consistent with proteomic data obtained from *Fusarium graminearum* and *Magnaporthe oryzae*, highly abundant Cu/Zn superoxide dismutase and small heat-shock molecular chaperones (sHSPs) were identified in the strain considered in this study. These proteins synergistically scavenge host ROS and maintain the correct folding of virulence enzymes under oxidative stress [51]. In addition, abundant ABC transporters were detected, which execute dual virulence functions—namely, efflux of plant antifungal substances and secretion of fungal toxic metabolites into host apoplasts—consistent with functional reports on numerous phytopathogens [52].

In addition to signaling and detoxification systems, secreted CAZymes and small cysteine-rich proteins (SCRPs) are direct virulence effectors. CAZymes can break down plant cell wall polysaccharides and promote hyphal expansion in *A. adsurgens*, while SCRPs may suppress plant immune signaling [48,53]. Other factors—including melanin synthases, ROS-scavenging proteins, and P450-linked toxin synthesis enzymes—likely act in an infection cascade with CAZymes and effectors: enzymes breach physical barriers, effectors weaken immunity, antioxidant proteins counter ROS, and secondary metabolites increase tissue damage [22,54].

The combined signaling, toxin biosynthesis, and detoxification proteins in *A. gansuense* overlap with validated virulence modules of *A. brassicicola*, including SSK regulators, P450s, and ABC transporters [21]. However, a major difference exists: many virulence-related proteins are induced only after host contact in *A. brassicicola*, while they are already highly expressed under axenic growth conditions in *A. gansuense*. This constitutive expression may allow for rapid infection initiation and might reflect a distinct, persistent parasitic strategy rather than rapid host-killing necrosis.

In summary, these co-expressed kinases, secondary metabolic enzymes, antioxidant proteins, and transporters likely form a layered pathogenic system. Although core protein families are conserved within the genus, in *A. gansuense* many stress-response, CAZyme, and toxin-related proteins are pre-accumulated. This may underlie its persistent infection strategy and distinguish it from fast-growing necrotrophs such as *A. brassicicola*. As our study involved only in vitro proteomics and homology-based inference, these associations remain putative and should be verified through targeted genetic and infection studies.

### 4.4. Limitations and Future Perspectives

This study produced a broad proteome map of *A. gansuense*, but several limitations remain. First, all proteomic data were obtained from axenic vegetative mycelia only. We did not analyze conidia, germ tubes, infection structures, or in planta tissues. Many fungal proteins are stage-specific; for example, GRG family proteins are often enriched in dormant spores rather than vegetative hyphae. By sampling only one stage, we may have missed proteins important at other stages, including infection-specific proteins. This also affected the interpretation of poorly annotated proteins, such as the highly abundant uncharacterized GRG1 domain protein.

Second, functional annotations were assigned solely from database matches (UniProt, NCBI CDD, KEGG, InterPro), which provide predictions and not direct evidence. Many families, including GRG domain proteins, remain poorly characterized across fungi, even in model species. As *A. gansuense* grows slowly and produces low biomass, the possibility for orthogonal validation (qRT-PCR, PRM, knockouts) was limited in this study.

Third, as conventional bottom-up DIA measures peptides aggregated from all proteoforms of a gene product, we could not fully resolve splice isoforms or post-translational modifications. This limitation particularly affects the interpretation of small lineage-specific proteins such as GRG family members, whose physiological activity may be regulated via post-translational modifications which are undetectable with the current peptide-level profiling approach. 

Despite these limitations, this work provides three novel contributions. First, we established a dual protein extraction strategy for fungi with melanized cell walls, which is useful for hard-to-lyse filamentous fungi. Second, we constructed the first full-scale DIA proteome of *A. gansuense*, covering more than 5000 proteins and many lineage-specific small proteins. Third, we proposed a coordinated pathogenicity framework linking signaling, secondary metabolism, and stress detoxification, which can subsequently be experimentally tested.

The results also suggest practical applications. Core candidates (SSK1p, P450s, ABC transporters) are promising targets for antifungal intervention, and the proteome map can support screening for host resistance in *A. adsurgens*. To address the abovementioned gaps, future work will include time-series proteomics across developmental and infection stages, integration with transcriptomic and metabolomic analyses, and targeted assays (including PRM) for candidate virulence and uncharacterized proteins. We also plan to analyze fungal secretomes from infected plants to track CAZymes and effectors under real infection conditions. In addition, transient expression in *A. adsurgens* and knockout/complementation of candidate genes will be used to test their putative pathogenic roles under nutrient limitation, oxidative stress, and host-mimicking conditions. These approaches are expected to clarify the infection network of *A. gansuense* and support the development of strategies for targeted disease management.

## 5. Conclusions

This study constructed the first comprehensive whole-proteome reference map of *A*. *gansuense*, the pathogen causing yellow stunt and root rot of *A*. *adsurgens*. Using combined 2-DE and DIA quantitative proteomics, we identified a total of 5052 proteins from vegetative mycelia. Multi-dimensional functional annotation analyses revealed that the proteome is dominated by proteins involved in primary metabolism, signal transduction, secondary metabolism, and stress response. Homology-based screening retrieved multiple putative virulence-related proteins, including two-component response regulators, protein kinases, cytochrome P450s, and ABC transporters. All these candidates are only bioinformatic predictions, requiring further in planta or genetic functional verification. Our annotation results indicate that these co-expressed proteins may form a coordinated functional module supporting vegetative growth and environmental stress tolerance in this fungus. Whether this protein module participates in the host–pathogen interaction remains unconfirmed and requires systematic follow-up functional testing. This baseline proteomic dataset provides a fundamental reference for future pathogenic mechanism research and supplies candidate molecules potentially enabling the development of novel control approaches for this damaging forage pathogen.

## Figures and Tables

**Table 1:** Normalized relative abundances of the top 20 most abundant proteins in *A. gansuense.*

Protein	Description	Gene	LYZ0006
A0A4Q4NIN8	Uncharacterized protein	AA0117_g5113	47,251,600
A0A8J2N6W0	alcohol dehydrogenase	ALTATR162_LOCUS6347	27,821,700
A0A177DJQ0	Superoxide dismutase [Cu_Zn]	AA0117_g5184	26,217,400
A0A4Q4NHQ7	SHSP domain_containing protein	AA0117_g6114	26,064,500
A0A8J2N540	Translation initiation factor 5A_like N_terminal domain_containing protein	ALTATR162_LOCUS11362	22,863,300
J9Y4F0	NADP_dependent mannitol dehydrogenase	Mdh	22,674,300
A0A5B9Y2C1	Elongation factor 1_alpha	EF1a	21,992,700
A0A177DE20	Glyceraldehyde_3_phosphate dehydrogenase	CC77DRAFT_1011328	20,756,300
Q75WR5	1,3,8_naphthalenetriol reductase	Brn1	18,637,600
A0A177DIT0	Ubiquitin_40S ribosomal protein S31 fusion protein	AA0117_g5152	18,387,100
Q9HDT3	Enolase	ENO	14,214,000
A0AAD4NSP1	Nucleoside diphosphate kinase	G6011_03302	11,900,800
A0A8J2MY05	Actin	ALTATR162_LOCUS3195	11,823,000
A0A8J2I9P2	Glucose_methanol_choline oxidoreductase	ALTATR162_LOCUS9581	11,597,000
A0A177DX03	Pyruvate decarboxylase	CC77DRAFT_1016629	11,533,000
A0A4Q4R994	Protein kinase domain_containing protein	AA0113_g9426	11,370,200
A0AAD4ILG0	peptidylprolyl isomerase	G6011_01685	10,821,700
A0ABR3UXD0	Peptidyl_prolyl cis_trans isomerase	ACET3X_000403	10,751,900
A0A8J2N2X9	YjgF_like protein	ALTATR162_LOCUS6822	10,395,000
A0AAD4I9A1	ATP synthase subunit alpha	G6011_02157	10,352,800

Notes: Protein represents the accession number retrieved from the reference database; Description refers to the functional annotation of matched proteins; Gene indicates the name of the corresponding coding gene, which is left blank if no annotation is available. The column LYZ0006 shows the mean normalized DIA-LFQ intensity of three independent biological replicates. Raw DIA MS intensities were calculated via the LFQ algorithm in DIA-NN, then median-normalized across all samples; these normalized relative quantitative values are unitless and reflect the basal abundance level of each protein in vegetative mycelia.

## Data Availability

The mass spectrometry proteomics data have been deposited to the ProteomeXchange Consortium via the PRIDE partner repository with dataset identifiers IPX0017912000 and PXD080022. All other supporting data are available within the manuscript and Supplementary Materials.

## Supplementary Materials

The following supporting information can be downloaded at: https://www.mdpi.com/article/10.3390/cimb48080818/s1.
