# Supplementary material for: Comprehensive Proteomic Profiling of Alternaria gansuense Provides Insights into Candidate Virulence-Associated Proteins and Core Physiological Features
Source: Curr Issues Mol Biol. 2026 Aug 12;48(8):818. doi: 10.3390/cimb48080818 (PMC13510438; doi:10.3390/cimb48080818)

Comprehensive Proteomic Profiling of *Alternaria gansuense* Provides Insights into Candidate Virulence-Associated Proteins and Core Physiological Features

Table S1. Liquid chromatography elution gradient table

Time (min)	Flow rate (μL/min)	Mobile phase A (%)	mobile phase B (%)
0	2.5	96	4
0.2	1.3	96	4
0.3	0.8	92	8
0.5	0.8	92	8
14.2	0.8	77.5	22.5
21.1	0.8	65	35
21.5	2.5	45	55
21.5		Column Wash	
21.9	2.5	1	99
22.6	2.5	1	99
22.6		Stop Run	

Notes: Mobile phase A: 0.1% (v/v) formic acid in ultrapure water; Mobile phase B: 0.1% (v/v) formic acid in acetonitrile. The gradient program includes peptide loading, gradient elution separation, and column washing steps; the chromatographic run is terminated at 22.6 min.

Figure S1. Schematic workflow of the whole proteomic experiment. Three independent biological replicates of *A. gansuense* vegetative mycelia were prepared. Two protein extraction pipelines were established: (1) phenol–methanol–ammonium acetate precipitation and TCA–acetone precipitation were compared via 2-DE to screen the optimal gel separation protocol; (2) SDT lysis method was adopted exclusively for DIA quantitative proteomics due to excellent compatibility with tryptic digestion and LC-MS/MS. All raw MS data were processed by DIA-NN, followed by multi-dimensional functional annotation to screen candidate virulence proteins and characterize core physiological features.

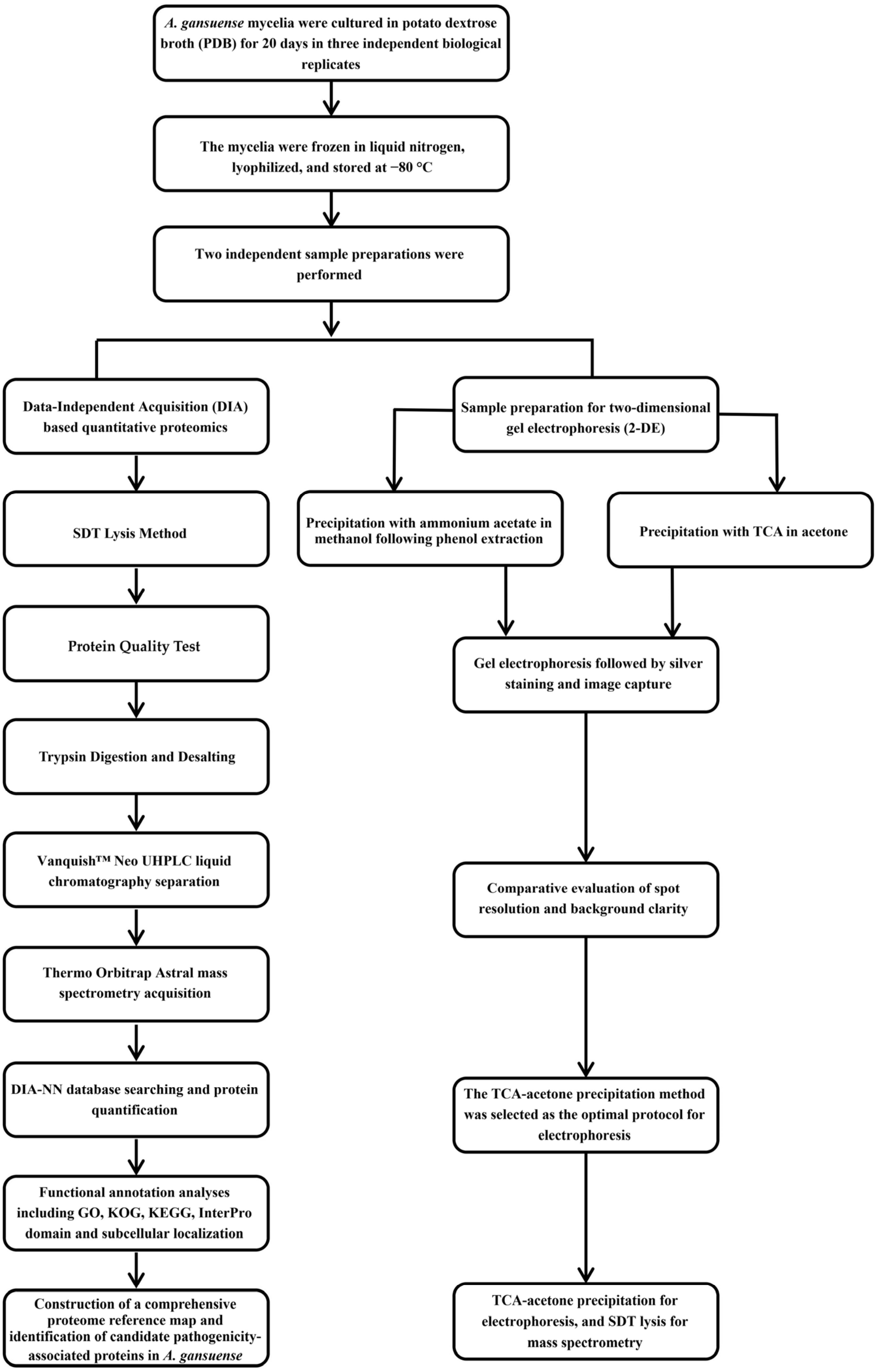

Figure S2. Length distribution of tryptic peptides identified by DIA-MS. Three biological replicates of mycelia were analyzed. Precursor intensities were normalized via global median normalization in DIA-NN. All peptide identifications were filtered at FDR ≤ 0.01, and peptide length statistics were exported directly from DIA-NN search results. The histogram was plotted using the OmicShare online platform.

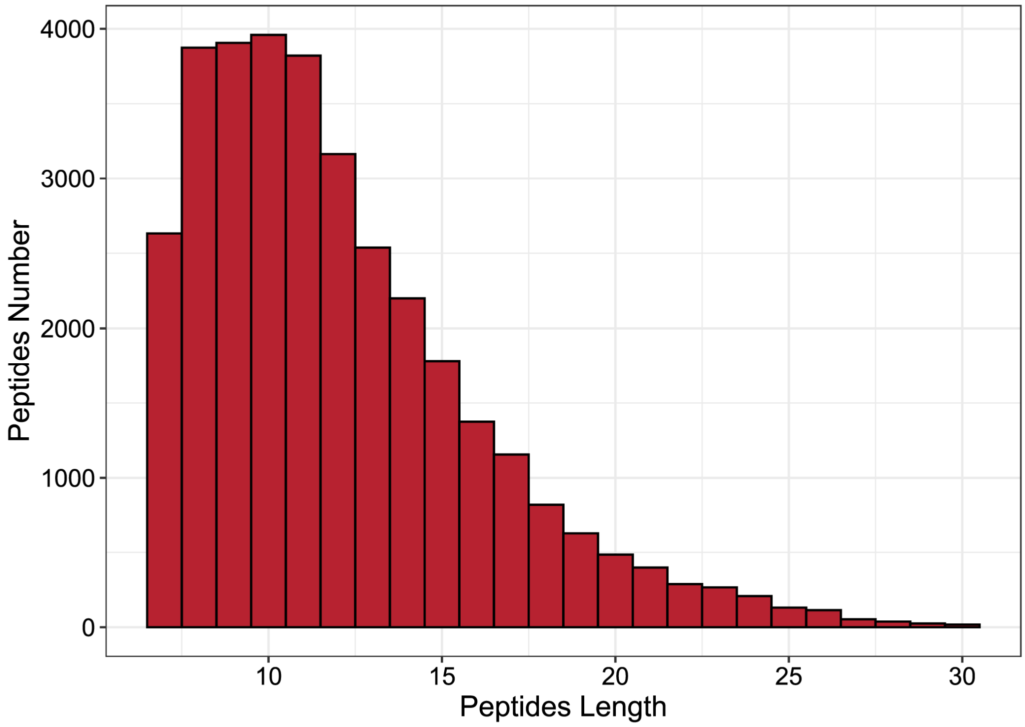

Figure S3. Cumulative distribution curve of unique peptide counts per identified protein. Most proteins were supported by at least one unique peptide, which guarantees reliable protein identification confidence under FDR ≤ 0.01 filtering.

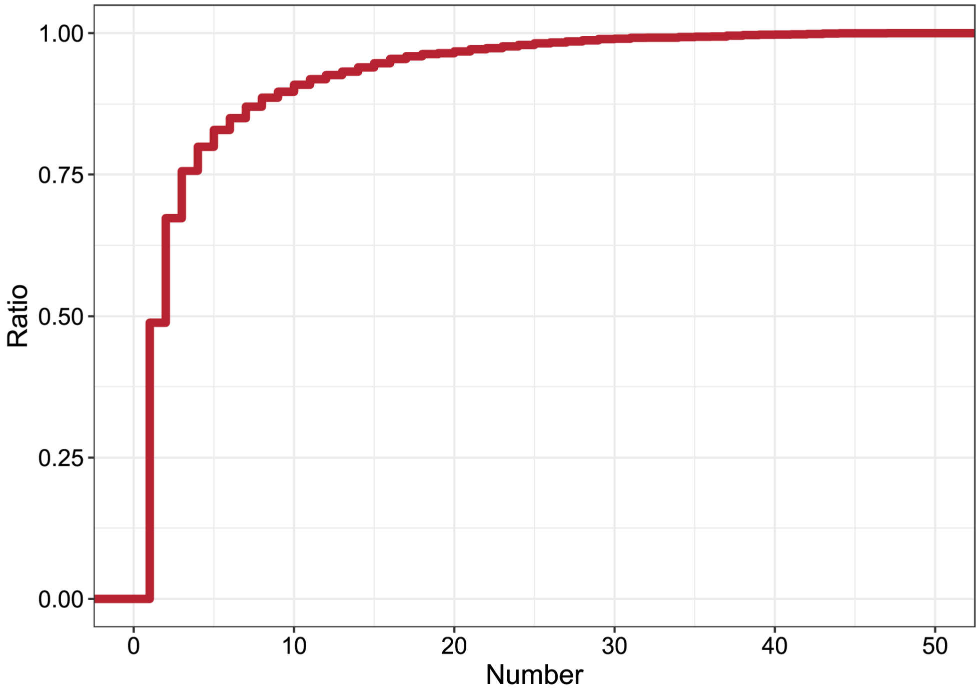

Figure S4. Retention time normalization distribution of all precursor ions from DIA-MS runs. The precursor retention deviations were concentrated near zero across all injections, demonstrating high stability of the UHPLC separation system.

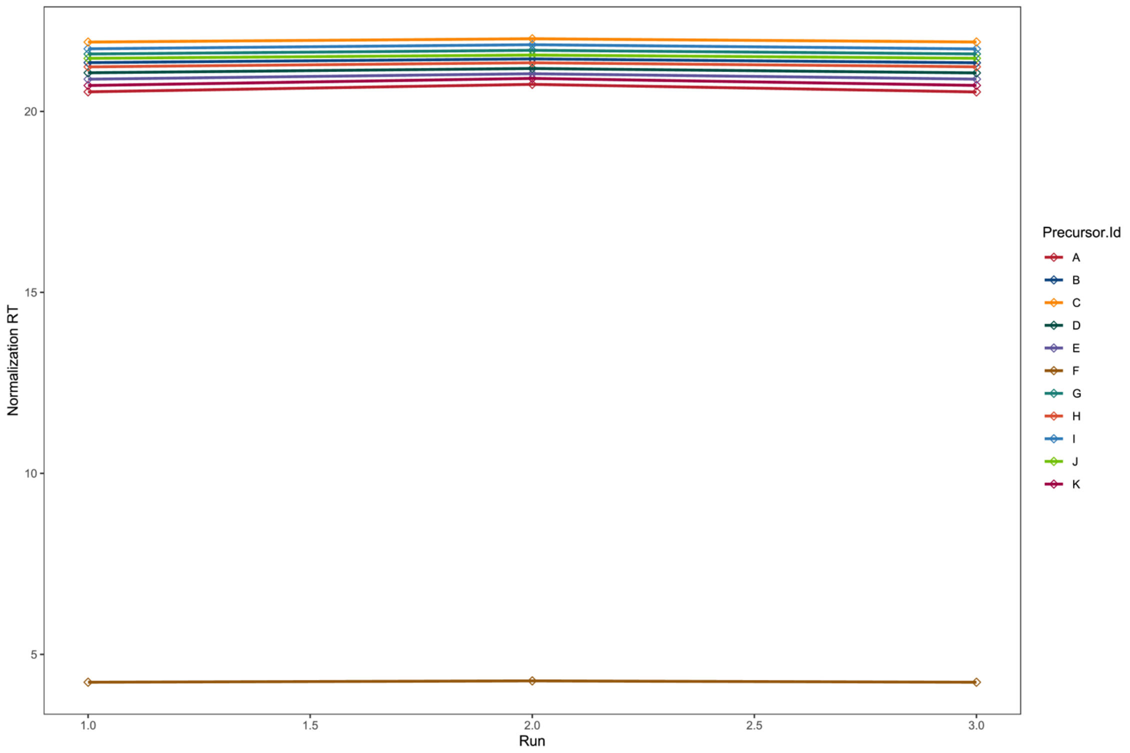

Figure S5. Pearson correlation heatmap of normalized protein abundance across three biological replicates. Correlation coefficients between any two samples were all higher than 0.92, indicating highly consistent global proteome profiles among parallel mycelial cultures. The color gradient corresponds to the magnitude of Pearson's R^2 value.

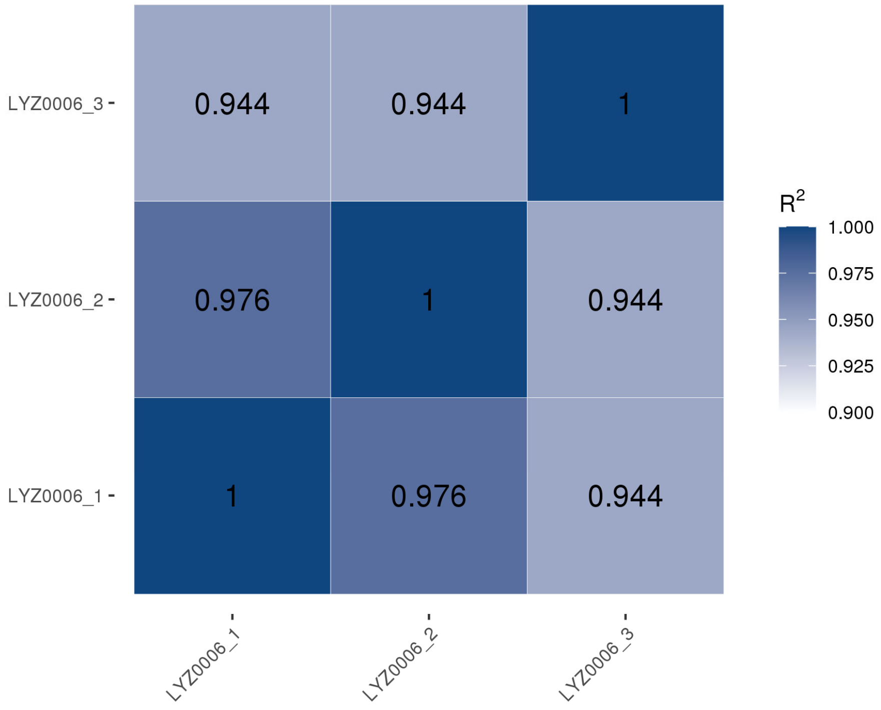

Figure S6. Principal component analysis (PCA) plot of three biological replicates with 95% confidence ellipse. All three mycelial samples clustered tightly within the confidence ellipse, reflecting negligible inter-batch biological variation and good reproducibility of the DIA dataset.

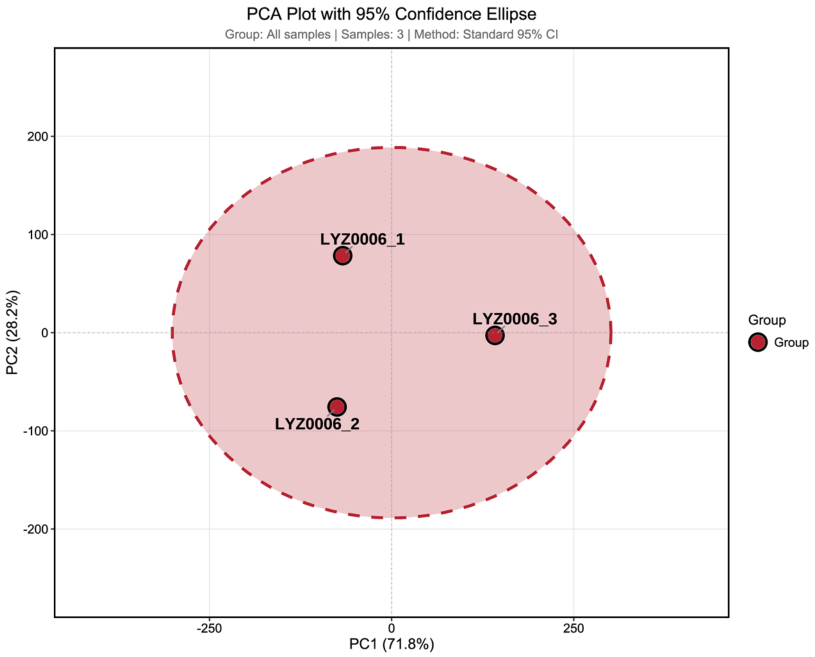

Figure S7. Cumulative distribution curve of coefficient of variation (CV) for all quantified proteins across three independent biological replicates. The X-axis represents CV values of normalized LFQ protein abundance, and the Y-axis indicates the cumulative proportion of quantified proteins. Statistical calculation revealed that 78% of all quantified proteins exhibited CV values lower than 0.2, which demonstrates satisfactory quantitative reproducibility among parallel mycelial cultures.

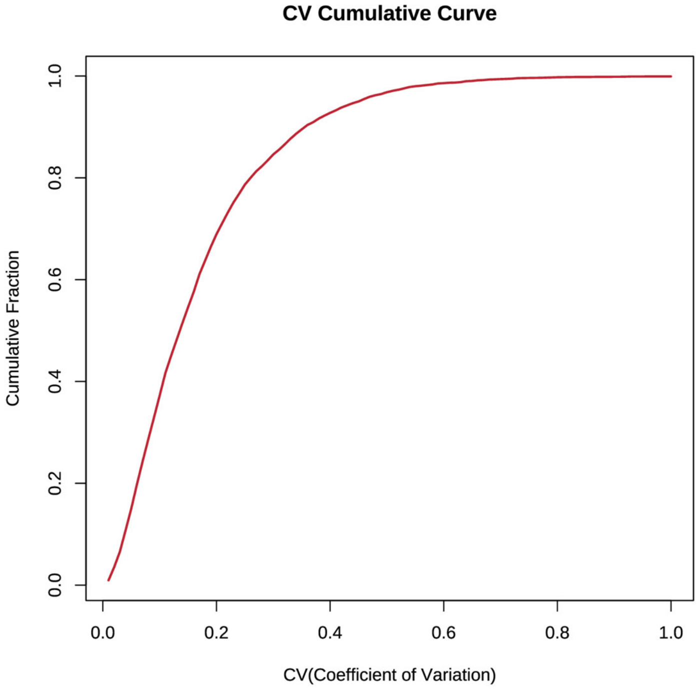

Supplement: Supplementary file 1 [file cimb-48-00818-s001.zip › cimb-4424914-supplementary.pdf]
